# Activated p38 MAPK in Peripheral Blood Monocytes of Steroid Resistant Asthmatics

**DOI:** 10.1371/journal.pone.0141909

**Published:** 2015-10-30

**Authors:** Ling-bo Li, Donald Y. M. Leung, Elena Goleva

**Affiliations:** 1 Department of Pediatrics, National Jewish Health, Denver, Colorado, United States of America; 2 Department of Pediatrics, University of Colorado Denver, Aurora, Colorado, United States of America; Faculty of Medicine & Health Sciences, UNITED ARAB EMIRATES

## Abstract

Steroid resistance is a significant problem in management of chronic inflammatory diseases, including asthma. Accessible biomarkers are needed to identify steroid resistant patients to optimize their treatment. This study examined corticosteroid resistance in severe asthma. 24 asthmatics with forced expiratory volume in one second of less then 80% predicted were classified as steroid resistant or steroid sensitive based on changes in their lung function following a week of treatment with oral prednisone. Heparinised blood was collected from patients prior to oral prednisone administration. Phosphorylated mitogen activated kinases (MAPK) (extracellular regulated kinase (ERK), p38 and jun kinase (JNK)) were analyzed in whole blood samples using flow cytometry. Activation of phospho-p38 MAPK and phospho-mitogen- and stress-activated protein kinase 1 (MSK1) in asthmatics’ peripheral blood mononuclear cells (PBMC) were confirmed by Western blot. Dexamethasone suppression of the LPS-induced IL-8 mRNA production by steroid resistant asthmatics PBMC in the presence of p38 and ERK inhibitors was evaluated by real time PCR. Flow cytometry analysis identified significantly stronger p38 phosphorylation in CD14^+^ monocytes from steroid resistant than steroid sensitive asthmatics (p = 0.014), whereas no difference was found in phosphorylation of ERK or JNK in CD14^+^ cells from these two groups of asthmatics. No difference in phosphorylated p38, ERK, JNK was detected in CD4^+^, CD8^+^ T cells, B cells and NK cells from steroid resistant vs. steroid sensitive asthmatics. P38 MAPK pathway activation was confirmed by Western blot, as significantly higher phospho-p38 and phospho-MSK1 levels were detected in the PBMC lysates from steroid resistant asthmatics. P38 inhibitor significantly enhanced DEX suppression of LPS-induced IL-8 mRNA by PBMC of steroid resistant asthmatics. This is the first report demonstrating selective p38 MAPK pathway activation in blood monocytes of steroid resistant asthmatics, suggesting that p38 and MSK1 phosphorylation can serve as blood biomarkers of steroid resistance.

## Introduction

Glucocorticoids (GCs) are potent anti-inflammatory drugs used for treatment of asthma and other inflammatory diseases. However, a number of patients are refractory to GC therapy[1, 2]. It is estimated that up to 20% of asthmatics do not respond to GCs, these patients are referred to as steroid resistant (SR) asthmatics[3]. SR asthmatics are characterized by increased airway inflammation that cannot be suppressed by GC treatment. The role of race, smoking, obesity, vitamin D level, allergens, and infection in steroid resistance is under active investigation[4–6].

Endotoxin exposure has recently been identified as an important factor that alters cellular response to GCs[7–9]. Our research group recently demonstrated alterations in airway microbiome of SR asthma patients, with the expansion of Gram-negative LPS producing bacteria[10]. We also reported significant levels of endotoxin in the bronchoalveolar lavage (BAL) fluid of SR asthmatics[8, 10]. Along with high endotoxin levels in BAL fluid, BAL macrophages of these patients demonstrated classical macrophage activation and induction of LPS signaling pathways[8]. Stimulation with LPS has been shown to result in the phosphorylation and activation of p38, ERK and JNK in monocytes and macrophages[11, 12]. Several studies have demonstrated that mitogen activated protein kinase (MAPK) pathways are involved in activation of transcription factors, such as NF-κB and AP-1[13, 14]; these transcription factors play a critical role in LPS-induced expression of proinflammatory genes, such as TNF-α, IL-1β, IL-6, IL-8, MCP-1, E-selectin, VCAM-1 and ICAM-1.

Cytoplasmic glucocorticoid receptor (GCR) mediates cellular response to GCs. Activated GCR translocates to the cell nuclei and acts as a transcriptional factor. GCR can inhibit pro-inflammatory MAPK signaling by inducing nuclear mitogen activated kinase phosphatase (MKP1) expression[15, 16]. At the same time, GCR activity is subject to kinase modulation, activated MAPKs can inhibit GCR function via phosphorylation that will inhibit GCR nuclear translocation in response to GC treatment, cause the GCR to return to the cytoplasm or modify GCR transcriptional activity[17, 18]. In this manuscript, we evaluated evidence for MAPK activation in peripheral blood of SR and SS asthmatics and asked whether MAPK activation in peripheral blood can serve as a biomarker of SR asthma.

## Materials and Methods

### Patients

We enrolled 24 adult asthma patients with airflow limitation (baseline FEV_1_≤80% predicted) and either airway hyperresponsiveness (PC_20_ methacholine < 8mg/ml) or bronchodilator responsiveness (>12% improvement in FEV_1_% predicted after 180 mcg metered-dose inhaler albuterol). Corticosteroid response of asthmatics was classified based on their prebronchodilator morning FEV_1_% predicted response to a one week course of 40mg/day oral prednisone. Asthmatics were defined as SR if they had less than 10% improvement in FEV1 and steroid sensitive (SS) if they showed significant improvement (≥12%). Informed written consent was obtained from all patients before enrollment in this study. The Institutional Review Board at National Jewish Health, Denver, Colorado approved this study. Research has been conducted according to the principles expressed in the Declaration of Helsinki. Subject characteristics are presented in **Table 1**.

**Table 1:** Patient characteristics*.

	SR asthma	SS asthma
	n = 13	n = 11
**Age, yrs**	38.5±3.3	41.4±4.0
**Gender (Male/Female)**	6/7	3/8
**Race (C/AA/Other)**	8/5/0	9/0/2
**BMI, kg/m** ^**2**^	30.8±2.3	32.4±2.6
**IgE, U/ml**	195±84	115±28
**Baseline FEV**_**1**_ **% predicted**	73.7±2.4	62.1±5.5
**FEV**_**1**_ **% reversal with Albuterol**	16.6±3.3	37.0±9.8
**FEV**_**1**_ **% change after Prednisone burst**	1.5±1.6*	39.8±10.6
**Medications** *** **: ICS/LABA**	4	4
**Medications** *** **: ICS**	3	2
**Medications: None**	6	5

*The values shown are Mean±SE.

**p<0.001 as compared to SS asthmatics.

***For the SR and SS asthmatics that received ICS/LABA or ICS the Mean±SE of the ICS dose in budesonide equivalents was 1163±402 μg and 1100±336 μg, respectively.

### Reagents and antibodies

Phosflow Lyse/Fix buffer, Phosflow Perm/Wash Buffer Ι and Stain Buffer were purchased from BD Pharmingen (San Diego, CA). Primary fluorophore-conjugated antibodies specific to phosphorylated proteins: mouse anti-p38 MAPK (pT180/pY182) Alexa 647 (cat. #612595) and mouse anti-p44/42 MAPK(pT202/pY204) Alexa 647 (cat. #612593), mouse IgG1 Alexa 647 (cat. #557783) were purchased from BD Pharmingen (San Diego, CA); mouse antibody against phosphorylated JNK (T183/Y185) Alexa 647 (cat. #9257) was from Cell Signaling Technology (Beverly, MA). Fluorophore-conjugated antibodies against cell-specific surface proteins: CD3 FITC, CD4 PE, CD8 PerCp-Cy5.5, CD14 FITC, CD16 PE, CD20 PerCP-Cy5.5 were purchased from BD Pharmingen. Anti-total and phosphorylated rabbit monoclonal antibodies to p38 MAPK (cat. #8690 and #4511), p44/42 MAPK (cat. #4695 and #4370), JNK (#4672 (rabbit polyclonal antibody) and #4668) and MSK1 (#3489 and #9595 (rabbit polyclonal antibody)) antibodies for Western blot analysis, as well as anti-Hsp90 (#4877) antibody were purchased from Cell Signaling Technology.

### Peripheral Blood Mononuclear Cell (PBMC) preparation

Heparinized peripheral blood was collected from all study subjects. Whole blood samples were used for flow cytometry analysis. PBMC were isolated by Ficoll-Hypaque® density gradient centrifugation. Isolated cells were resuspended at 1x10^6^ cells/ml in RPMI 1640 medium containing 10% charcoal-filtered fetal calf serum (Gemini Bio-Products, Calabasas, CA).

### Cell surface and intracellular phospho-MAPK flow cytometry analysis

300μl of blood was fixed in 6 ml of BD Phosflow Lyse/Fix buffer at 37°C for 10 min and then washed once with PBS. After centrifugation, the cell pellet was permeabilized in BD Phosflow Perm/Wash Buffer Ι for 10 min at room temperature. Cell pellet was resuspended in 200μl of staining buffer, and cell suspension was equally divided between four FACS tubes containing 50μl of monoclonal antibodies (CD surface antibodies plus anti-phospho-MAPK or isotype control). Cells were incubated at room temperature for 45 min in the dark. After the final wash, cells were resuspended in 1% paraformaldehyde (200μl) and stored at 4°C. Cells were examined using FACScaliber (San Jose, CA) and data were analyzed using CellQuest software.

We tested the levels of total MAPK expression in several cell types from SR and SS asthma patients by flow cytometry. We did not observe the difference in total MAPK expression between the cells from two study groups (data not shown). Therefore we chose only to assess MAPK phosphorylation status by flow cytometry.

### Western blot

Whole cell extracts were prepared from freshly isolated PBMC using the ice-cold complete lysis buffer (1x Cell Signaling Lysis Buffer, #9803, Cell Signaling Technology, Inc., Danvers, MA)) with 10μL each PMSF, protease inhibitor cocktail, and sodium orthovanadate (#sc-24948, Santa Cruz Biotechnology, Inc., Santa Cruz, CA) per 1ml of lysis buffer). Cells were lysed for 5 min, scraped of the plates, sonicated for 10s and spun for 10 min at 10,000g at 4°C. 50μg of protein per condition were run on a 4–25% gradient gel (Bio-Rad Laboratories, Hercules, CA) and transferred to nitrocellulose membranes. The membranes were blotted with 1:1000 primary antibodies overnight at 4°C. After serial washes with TBS containing 0.1% Tween 20, membranes were incubated with appropriate peroxidase-conjugated secondary antibodies for 1 h at room temperature. Immunoreactive bands were visualized using a chemiluminescent kit (GE Healthcare) and images were captured on film (GE Healthcare). Developed X-ray films were scanned and densitometry of the bands were quantified with NIH Image software (version 1.63) (this software is available on the Internet at http://rsb.info.nih.gov/nih-image). Hsp90 detection was used to control the quality of protein preparation and to ensure that equal amounts of cellular proteins were loaded per lane.

### LPS-induced cytokine production by PBMC in the presence of MAPK inhibitors and corticosteroids

PBMC were resuspended in RPMI 1640 (BioWhittaker) containing 10% charcoal filtered heat inactivated steroid-free FCS (Gemini Bio Products, Calabasas, CA), 40 μmol/l L-glutamine, 100 U/ml penicillin, 100 U/ml streptomycin, and 20 mmol/l HEPES (GIBCO BRL Life Technologies) at a concentration of 1×10^6^/ml, pretreated with 1μM p38 MAPK inhibitor (SB203580) or with 10μM ERK inhibitor (PD98059) for 1h, followed by incubation with 10 ng/ml LPS in the absence or presence of 10^-7^M dexamethasone (DEX). Cells were collected after 24h of incubation and IL-8 mRNA expression was examined by real time PCR.

### Real time PCR (RT-PCR)

Total RNA was prepared using RNeasy Mini kit (Qiagen, Valencia, CA). After reverse transcription, 500 ng cDNA from each sample were analyzed by RT-PCR using the dual-labeled fluorogenic probe method on an ABI Prism 7300 RT-PCR system (Applied Biosystems). The expression of human IL-8 mRNA and 18s RNA was determined. All primers were purchased from Applied Biosystems (Foster City, CA).

### Statistical analysis

Results were expressed as the Mean±SE. Statistical analysis was conducted using GraphPad Prism, version 5 (GraphPad Software, La Jolla, CA). Unpaired Student’s *t* test and nonparametric tests were used. P<0.05 were considered significant.

## Results

### p38 phosphorylation in PBMC of asthmatics

Previous studies have documented p38 MAPK activation in BAL macrophages of severe asthma patients[19, 20]. Given our recently reported data on classical macrophage activation in BAL macrophages of SR asthmatics[8] and evidence for the expansion of LPS producing bacteria in the airways of SR asthmatics,[10] we evaluated whether MAPK activation can be observed in the peripheral blood of SR asthmatics as compared to SS asthmatics. Patients were defined as SR or SS based on changes in lung function after one week of oral prednisone burst. Asthma patients recruited had airway obstruction (**Table 1**). Despite bronchodilator responsiveness, SR asthma patients did not demonstrate improvement in lung function after oral prednisone burst, while SS patients were bronchodilator responsive and significantly improved their lung function after one week of oral prednisone treatment (**Table 1**). Western blot analyses were performed using whole protein extracts prepared from PBMC of SR and SS asthmatics.

**Fig 1:**
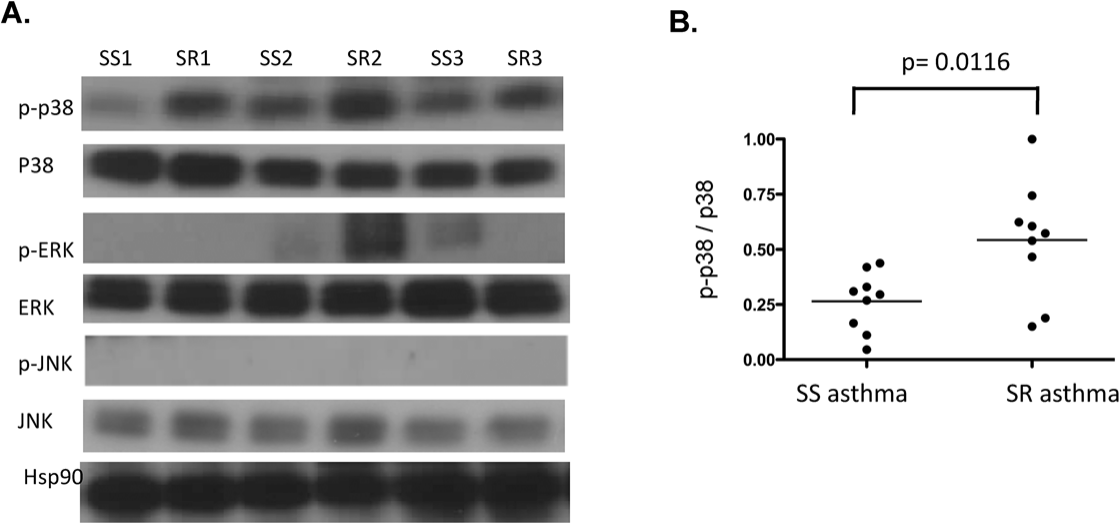
MAPKs phosphorylation in freshly isolated PBMC of SS asthmatics and SR asthmatics as assessed by Western blot. **(A)** Cell lysates were prepared from the PBMC of nine subjects with SS asthma and nine subjects with SR asthma. 50 μg of protein from each patient PBMC cell extract were run on a 4–20% gradient gel and blots were probed with antibodies against phosphorylated forms of p38, ERK and JNK. Membranes were stripped and re-probed with antibodies against total p38, ERK and JNK, respectively. Hsp90 detection was used to monitor protein loading. **(B)** Densitometry values of phosphorylated form of p38 to total p38 in PBMC of SR and SS asthmatics.

We observed presence of the activated form of p38, phosphorylated p38 MAPK (p-p38) in PBMC of all subjects with asthma (**Fig 1**). Moreover, significantly increased phosphorylation of p38 MAPK was detected in PBMC extracts from SR asthmatics compared with SS asthmatics (p<0.0116). The expression of total p38, however, in cells of the two groups of asthmatics was similar.

In contrast, phosphorylated ERK was found in the cell lysates of only one SR asthma subject out of 18 asthmatics tested. No JNK phosphorylation was detected in PBMC extracts from 18 asthmatics examined. The activity of anti-phospho-JNK antibody was validated with the lysates from LPS stimulated PBMC (data not shown).

### Increased p38 phosphorylation in peripheral blood CD14^+^ monocytes of SR asthmatics

We further asked what specific subsets of PBMC contribute to the increased p38 phosphorylation in PBMC of SR asthmatics. At the same time, ERK and JNK activation in PBMC was evaluated, as it was possible that activation of these kinases was only present in specific rare cell subsets in PBMC and therefore could not be detected by Western blot analysis using the PBMC extracts.

**Fig 2:**
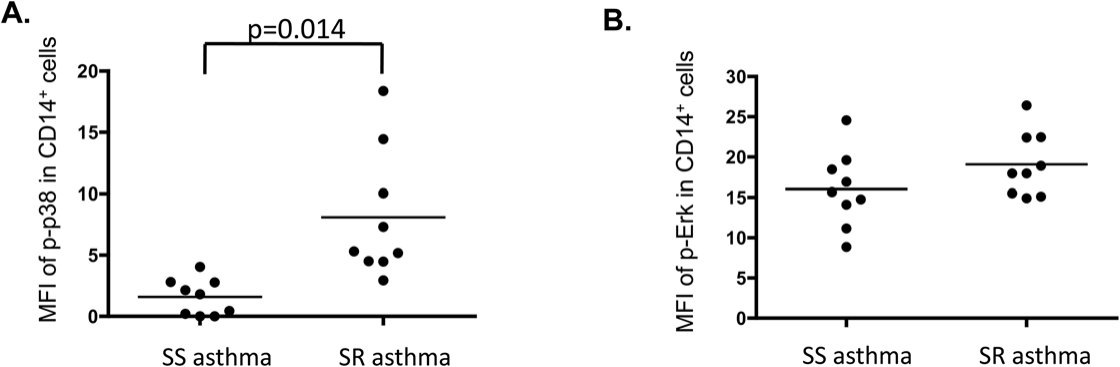
Flow cytometry assessment of MAPK phosphorylation in whole blood samples of SR and SS asthma patients. Whole blood aliquots were stained with either anti-CD3, CD4, CD8 antibody cocktail or CD14, CD16, CD20 antibody cocktail plus one of the phospho-MAPK antibodies (i.e. p-p38, p-ERK, p-JNK) or corresponding IgG isotype control antibodies (to detect nonspecific antibody binding). **(A)** Phosphorylation of p38 in CD14^+^ monocytes of subjects with SR asthma vs. SS asthma (MFI). (**B**) Phosphorylation of ERK in CD14+ cells of subjects with SR asthma vs. SS asthma (MFI).

To do this, we analyzed p38, ERK and JNK phosphorylation by flow cytometry using heparinized whole blood samples from asthmatics. We discovered that there was a significantly stronger signal for activated p38 in CD14^+^ monocytes from SR than SS asthmatics (p = 0.014) (**Fig 2A**), whereas no difference in phosphorylation of ERK and JNK in CD14^+^ cells was observed in the two groups of asthmatics (**Fig 2B**). In addition, no significant difference in phosphorylated p38, ERK, or JNK was detected in CD4^+^, CD8^+^ T cells, B cells and NK cells from SR vs. SS asthmatics (**Table 2**).

**Table 2:** MFI of phosphorylated MAPK staining in various cell types in the blood samples of asthmatics.

Cell type	MFI p-ERK		MFI p-p38		MFI p-JNK	
	SS asthma	SR asthma	SS asthma	SR asthma	SS asthma	SR asthma
**CD3+CD4+ (T helper cells)**	1.26±0.31	1.29±0.40	1.18±0.31	0.81±0.23	7.34±1.51	8.07±1.41
**CD3+CD8+ (T cytotoxic cells)**	1.83±0.85	1.95±0.86	0.95±0.34	1.83±0.85	4.24±1.30	3.75±1.04
**CD14-CD16+ (NK cells)**	1.75±0.48	1.84±0.39	2.63±0.73	1.74±0.48	4.06±0.86	3.96±0.59
**CD14-CD20+ (B cells)**	0.38±0.09	0.68±0.18	1.30±0.37	0.79±0.27	1.56±0.39	0.86±0.25

### Evidence for p38 pathway activation in cells from SR asthmatics

**Fig 3:**
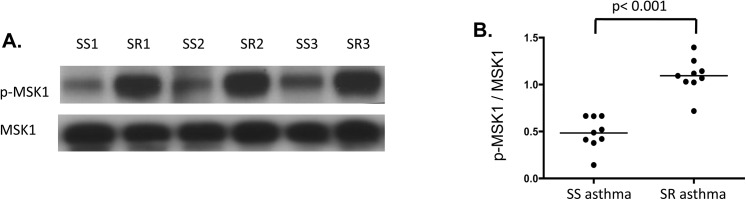
Activation of the p38 pathway in the cells of SR asthmatics. **(A)** Significantly elevated p-MSK1 levels in lysates from PBMC of SR as compared to SS asthma patients. Representative Western blot results of p-MSK1 and MSK1 in PBMC are shown. PBMC protein lysates prepared to assess p38 phosphorylation **(Fig 1)** were examined for p-MSK1 expression. **(B)** Densitometric values of phosphorylated form of MSK1 to total MSK1 in PBMC of SR and SS asthmatics.

To support the importance of p38 activation in the cells of SR asthmatics, we evaluated phosphorylation of mitogen- and stress-activated kinase 1 (MSK1), a kinase directly downstream of p38. MSK1 phosphorylation was examined by Western blot using the same cell lysates prepared to assess p38 phosphorylation in the study subjects. The levels of MSK1 phosphorylation were found to be significantly higher in PBMC lysates from SR asthmatics as compared to SS asthmatics (**Fig 3A and 3B**), p< 0.001.

### Effects of p38 MAPK inhibitor on responses of cells from SR asthmatics to GCs

**Fig 4:**
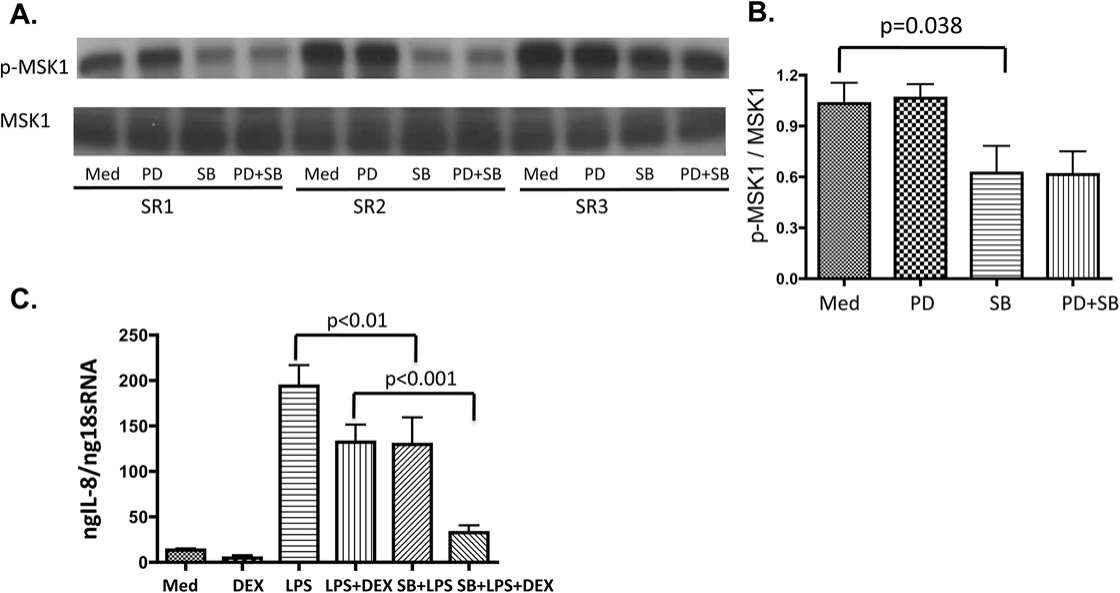
Inhibition of p38 MAPK activation using p38 inhibitor SB203580 results in increased DEX suppression LPS-induced IL-8 mRNA production. P38 MAPK inhibition by p38 inhibitor SB203580 (**SB**), but not ERK inhibitor, PD98059 (**PD**) results in selective inhibition of downstream kinase activation (MSK1 phosphorylation) in the cells of SR asthmatics as shown by Western blot (**A**, **B**). After preincubation of PBMC from SR asthmatics with a selective p38 inhibitor the cells became steroid sensitive, i.e. greater suppression of LPS induced IL-8 mRNA by DEX, as shown by RT-PCR (**C**).

To evaluate the role of p38 activation in steroid resistance, PBMC from SR asthmatics were preincubated with a selective p38 MAPK inhibitor, SB203580. As previously reported[21], p38 MAPK remains phosphorylated after treatment with SB203580, but its catalytic activity is inhibited. Phosphorylation of the downstream kinase MSK1 was chosen as a read out for p38 suppression by SB203580. MSK1 phosphorylation was significantly decreased in the cells of SR asthmatics pretreated with p38 MAPK inhibitor, but not with the ERK inhibitor, PD98059 (**Fig 4A and 4B**). Importantly, cells of SR asthmatics that were pretreated with the p38 inhibitor became significantly more responsive to the suppressive effects of GCs. As shown by RT-PCR, preincubation of PBMC from SR asthmatics with SB203580 resulted in significantly greater DEX-mediated suppression of LPS induced IL-8 mRNA (**Fig 4C**).

## Discussion

Steroid resistance is a common problem in many chronic inflammatory diseases, including asthma. Our current study demonstrates that there is a selective increase in p38 activation in peripheral blood monocytes of SR asthma patients compared to the cells of SS asthmatics. This differential p38 activation was not observed in CD4^+^, CD8^+^ T cells, B cells or NK cells. The functional significance of p38 pathway activation in SR asthmatics was supported by our observation of a significant increase in MSK1 phosphorylation and lack of GC suppression of IL-8 mRNA production by the cells from SR asthmatics. Importantly, ERK and JNK phosphorylation in PBMC of SR asthmatics was not remarkable or negative. This data suggests that peripheral blood p38 MAPK pathway activation can serve as a selective biomarker for SR asthmatics.

In a previous study, we demonstrated the expression of multiple inflammatory genes related to classical macrophage activation in BAL cells from SR asthmatics as compared with SS asthmatics[8]. Activated ERK, JNK and p38 MAPK had been found in airway epithelial cells and alveolar macrophages of patients with asthma[20, 22, 23] and studies have demonstrated association between the clinical severity of asthma and expression of p-p38 in asthmatic airways[24]. P38 MAPK was also demonstrated to regulate the production of many pro-inflammatory cytokines such as IL-8, IL-6, TNF-α; increased levels of IL-8 and TNF-α have been found in BAL macrophages from SR asthmatics[24]. In a recent study, Bhavsar et al. stimulated alveolar macrophages from asthma patients of different severities with LPS and concluded that steroid responsiveness was correlated with the degree of p38 MAPK activation[19]. Our current study found enhanced p38 phosphorylation also in circulating CD14^+^ cells of SR asthmatics. To the best of our knowledge, this is the first report to compare the levels of phosphorylated MAPK in components in PBMC and the first study that demonstrates increased p38 phosphorylation in circulating blood CD14^+^ from patients with SR asthma as compared to SS asthma.

P38 consists of four subtypes (p38 alpha, beta, gamma and delta), which regulate cellular activities by serving as phosphorylation substrates of its upstream kinases, and also phosphorylates specific serines and threonines of its downstream substrates. Studies suggest that different p38 isoforms have overlapping, but also distinct physiological roles[25, 26]. The anti-p-p38 MAPK antibody used in this study recognizes all p38 MAPK isoforms. On the other hand, p38 MAPK inhibitor, SB203580, that was used in this study, is an inhibitor for p38 MAPK alpha and beta isoforms; p38 MAPK gamma and delta isoforms are insensitive to this class of pyridinyl imidazole p38 MAPK inhibitors[27]. Pretreatment of PBMC with SB203580 inhibited p38 MAPK activity in the cells and enhanced ability of DEX to suppress IL-8 production by PBMC. This suggests that phosphorylated p38 MAPK in the cells of SR asthma patients is likely an alpha/beta isoform rather then gamma/delta. A recent study demonstrated inhibition of p38 MAPK gamma isoform by formeterol in PBMC pretreated by IL-2/IL-4[28]. In our study we did not observe the difference in p38 MAPK activation between patients treated with ICS/LABA as compared to patients treated with ICS only (data not shown). Although steroids have been reported to inhibit p38 MAPK phosphorylation in airway epithelial cells,[29] we did not observe the difference in p38 MAPK activation between steroid users and non-steroid users in both SR and SS asthma groups (data not shown).

Downstream of p38, there is MSK1, which can mediate phosphorylation of histone H3 on serine at different inflammatory gene promoters to stimulate their transcription[30]. However, GCs counteract the recruitment of activated MSK1 at inflammatory gene promoters resulting in the inhibition of NF-κβ, p65 transaction and concurrent histone H3 phosphorylation[31]. Another example of interaction between the GCR and MSK1 is that MSK1 predominantly localizes in the nucleus, however, activated GCR can translocate p-MSK1 from the nucleus to the cytoplasm, therefore inhibiting MSK1 effects on its downstream targets[32]. In our current study, we detected an increase in activated MSK1 that paralleled the increase of phosphorylated p38 in PBMC from SR asthma, indicating the potential involvement of MSK1 in regulation of cellular steroid responses.

Our data is consistent with previous observations by several research groups that p38 MAPK inhibition in severe asthma may enhance cellular responsiveness to GCs[9, 19, 33, 34]. Several studies have also demonstrated effectiveness of p38 inhibition in the reversal of GC insensitivity of cells from COPD patients[35, 36]. However previous studies have not identified the cell type in peripheral blood with selective p38 MAPK activation in severe and/or steroid resistant asthma. Our study demonstrates for the first time p38 and MSK1 activation in peripheral blood monocytes of SR asthmatics. Peripheral blood from patients can be readily analyzed. Thus, p-p38 and p-MSK1 should be further evaluated in PBMC as potential biomarkers of asthmatic responsiveness to GC treatment.
